# P-1076. Describing the clinical characteristics, treatment patterns, and outcomes in hospitalized pneumonia patients treated with Ceftolozane/Tazobactam (C/T): Insights from the SPECTRA Study

**DOI:** 10.1093/ofid/ofae631.1264

**Published:** 2025-01-29

**Authors:** Emre Yucel, Alex Soriano, David Paterson, Florian Thalhammer, Stefan Kluge, Pierluigi Viale, Mike Allen, Brune Akrich, Yanbing Zhou, Huina Yang, Sundeep Kaul

**Affiliations:** Merck & Co., Inc., North Wales, Pennsylvania; Hospital Clínic de Barcelona, Barcelona, Catalonia, Spain; National University of Singapore, Singapore; Medizinische Universität Wien, Vienna, Wien, Austria; Department of Intensive Care, University Medical Center Hamburg-Eppendorf, Hamburg, Hamburg, Germany; Infectious Diseases Unit, Department of Medical and Surgical Sciences, Policlinico Sant'Orsola Malpighi, University of Bologna, Bologna, Italy, Bologna, Emilia-Romagna, Italy; MSD, UK, Ltd., London, England, United Kingdom; Merck Research Labs, MSD, Puteaux, Ile-de-France, France; Merck, Rahway, New Jersey; Tan Tock Seng Hospital, Singapore, Not Applicable, Singapore; Harefield hospital, london, England, United Kingdom

## Abstract

**Background:**

This sub-analysis of the SPECTRA (Study of Prescribing patterns and Effectiveness of Ceftolozane/Tazobactam [C/T] Real-world Analysis) study aimed to describe the clinical characteristics, treatment patterns, and outcomes of hospitalized pneumonia patients treated with ceftolozane/tazobactam (C/T).

**Table 1.**
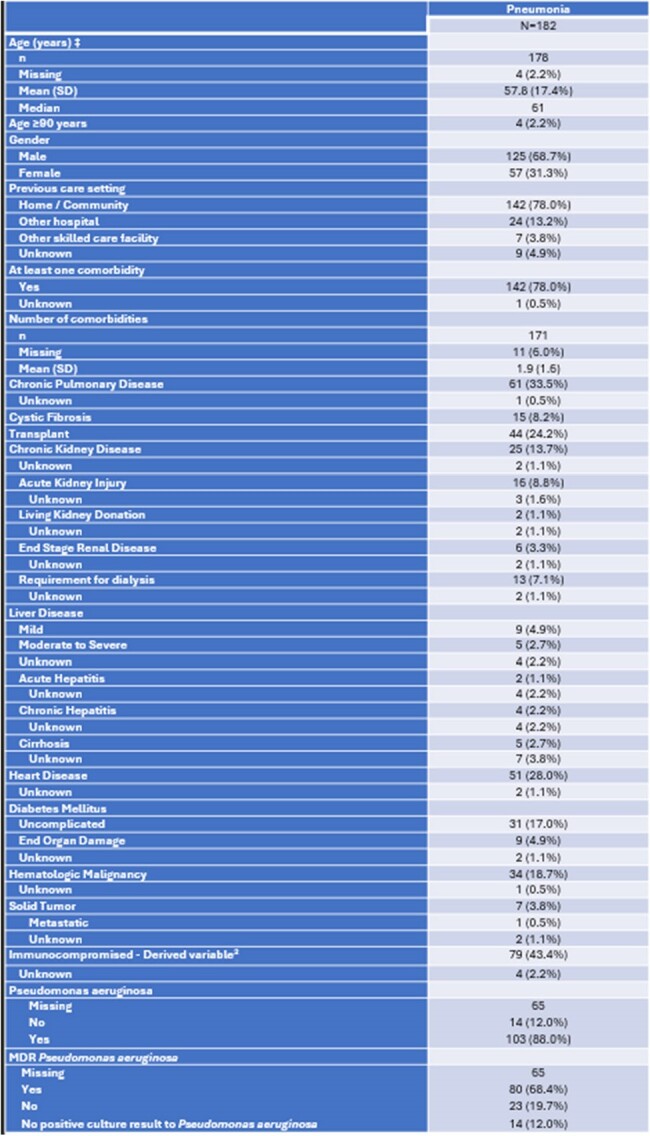
Patient characteristics and Microbiology 2 Immunocompromised defined as: ‘Immunocompromised present’ [raw item] or ‘Hematologic Malignancy present’ or ‘Solid Tumor present’ or ‘Transplant=Yes’.

**Methods:**

SPECTRA was a multicenter observational study conducted in 7 countries. It included adult patients (age≥18yrs) who received ≥48-hours C/T treatment (n=617). Retrospective data were extracted from medical records covering a 6-month period prior to the index date, up to 30 days after the last dose of C/T or until death. Descriptive statistics were used to analyze clinical success, all-cause in-hospital mortality (ACHM), intensive care unit (ICU) admission, length of stay (LOS) in the subcategory of pneumonia (n=182).

**Table 2.**
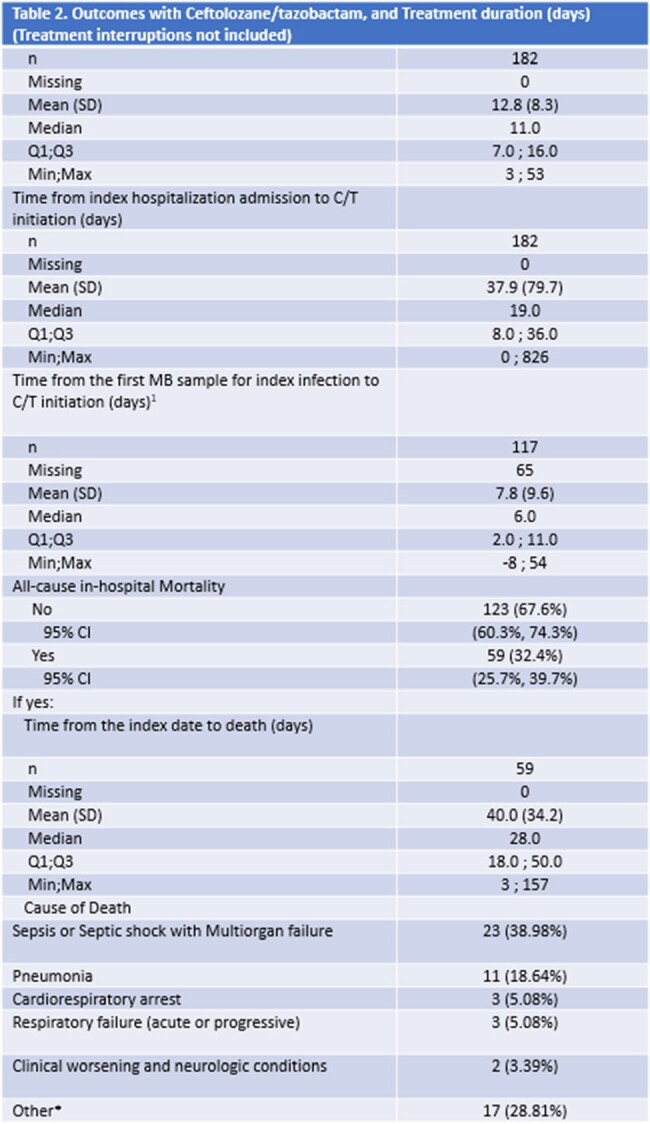
Outcomes with Ceftolozane/tazobactam and Treatment duration (days) (Treatment interruptions not included) *Other conditions: Bile duct anostomic leakage, Brain-vascular hemorrhagic accident, CMV infection, Encephalic Death, Hemorrhage, Invasive fungal infection, Necrotizing pancreatitis, Post operative complication, Renal amyloidosis, Spontaneous rupture of the spleen, Septic shock, Hemodynamic dysfunction, Kidney failure, Stable angina grade I-II CCS, Probable Clostridium difficile colitis, Progression of AML, Refractory Hypoxemia, Refractory hematologic malignancy, Subarachnoid Hemorrhage, Solid tumor, Tracheobronchitis

**Results:**

The median duration of C/T treatment was 11.0 days, median time from index hospitalization admission to C/T initiation was 19.0 days, and from the first microbiological sample to C/T initiation 6.0 days. All-cause in-hospital mortality was 32.4% (n=59), with a median time from the index to death of 28.0 days. The causes of death included sepsis (38.9%) and pneumonia (18.6%) and 67% (n=122) patients were admitted to the ICU during the index hospitalization. Pseudomonas aeruginosa (PSA) was the most commonly isolated pathogen (88% of patients), with MDR PsA present in 68.4% (n=80) of pneumonia patients.

**Conclusion:**

In hospitalized patients with pneumonia treated with C/T in a real-world setting, clinical characteristics, treatment patterns, and outcomes provide insights into the use and effectiveness of C/T.

**Disclosures:**

**Emre Yucel, PhD**, Merck: I am a full time Merck Employee and own stocks in the retirement plan provided by Merck.|Merck: Stocks/Bonds (Public Company) **David Paterson**, bioMerieux: Grant/Research Support|bioMerieux: Honoraria|Merck: Advisor/Consultant|Merck: Grant/Research Support|Merck: Honoraria|Pfizer: Advisor/Consultant|Pfizer: Grant/Research Support|Pfizer: Honoraria|Shionogi: Grant/Research Support|Shionogi: Honoraria **Florian Thalhammer, MD**, MSD: Advisor/Consultant **Stefan Kluge, Prof. Dr. med.**, Merck & Co: Advisor/Consultant|Merck & Co: Board Member **Mike Allen, PhD**, Merck: I am a full time Merck Employee and own stocks in the retirement plan provided by Merck.|Merck: Stocks/Bonds (Public Company) **Brune Akrich, MD**, Merck: I am a full time Merck Employee and own stocks in the retirement plan provided by Merck.|Merck: Stocks/Bonds (Public Company) **Yanbing Zhou, PhD**, Merck: I am a full time Merck Employee and own stocks in the retirement plan provided by Merck.|Merck: Stocks/Bonds (Public Company)

